# The Role of Avocados in Complementary and Transitional Feeding

**DOI:** 10.3390/nu8050316

**Published:** 2016-05-21

**Authors:** Kevin B. Comerford, Keith T. Ayoob, Robert D. Murray, Stephanie A. Atkinson

**Affiliations:** 1 Department of Nutrition, University of California at Davis, Davis, CA 95616, USA; 2 Department of Pediatrics, Albert Einstein College of Medicine, Bronx, NY 10461, USA; keith.ayoob@einstein.yu.edu; 3 Department of Human Sciences, The Ohio State University, Columbus, OH 43210, USA; murrayMD@live.com; 4 Department of Pediatrics, McMaster University, Hamilton, ON L8S 4KI, Canada; satkins@mcmaster.ca

**Keywords:** avocado, monounsaturated fat, fiber, infant, toddler complementary feeding, transitional feeding

## Abstract

Infant dietary patterns tend to be insufficient sources of fruits, vegetables, and fiber, as well as excessive in salt, added sugars, and overall energy. Despite the serious long-term health risks associated with suboptimal fruit and vegetable intake, a large percentage of infants and toddlers in the U.S. do not consume any fruits or vegetables on a daily basis. Since not all fruits and vegetables are nutritionally similar, guidance on the optimal selection of fruits and vegetables should emphasize those with the greatest potential for nutrition and health benefits. A challenge is that the most popularly consumed fruits for this age group (*i.e.*, apples, pears, bananas, grapes, strawberries) do not closely fit the current general recommendations since they tend to be overly sweet and/or high in sugar. Unsaturated oil-containing fruits such as avocados are nutritionally unique among fruits in that they are lower in sugar and higher in fiber and monounsaturated fatty acids than most other fruits, and they also have the proper consistency and texture for first foods with a neutral flavor spectrum. Taken together, avocados show promise for helping to meet the dietary needs of infants and toddlers, and should be considered for inclusion in future dietary recommendations for complementary and transitional feeding.

## 1. Introduction

Proper nutrition is one of the most influential factors for insuring normal growth and development during a child’s first years of life, yet there are currently very few research-based dietary recommendations for parents and caregivers regarding this critical time period [1]. Currently, U.S. dietary guidelines do not differentiate between age groups of children under two years old even though the nutritional requirements of infants and toddlers differ from each other, and from the nutritional requirements of older children, adolescents, and adults. Without age-appropriate dietary guidelines from birth to 24 months of age, the diets of infants and toddlers often reflect the dietary preferences and nutrient composition of their parents’ and caregivers’ diets, which tend to lack adequate sources of fiber-rich and nutrient-dense fruits and vegetables and contain excessive sodium and sugar [1].

Nutrition delivery to infants is influenced by physiological development, growth demands, and to some extent by family dietary practices. During the first six months after birth, breast milk is recommended as the exclusive source of nutrition necessary for early infant health and development [2], especially because during the early stages of development, a baby’s swallowing and digestive systems are not yet fully developed for the intake and processing of non-liquid food sources [3]. As an infant grows and develops, it experiences physiological shifts in nutrient and energy requirements that can no longer be supported by breast milk alone [2]. Additionally, as an infant transitions from a strictly maternal-sourced food supply to a varied diet and familial table foods, the emphasis on energy and nutrient requirements is placed on a whole new set of dietary options—many of which are not ideal for young children.

Although the timeframes for complementary and transitional feeding are not officially defined, for this review, the complementary feeding period is considered to start when the first foods (other than breast milk or formula) are consumed and continues throughout the infant’s first year of life (approximately six to 12 months), while the transitional feeding period covers a toddler’s dietary pattern during their second year of life (13–24 months). Nutrient and energy deficiencies during the early developmental stages can have lasting effects on the health of the offspring [4,5,6]. Therefore, nutrient-rich foods that are also moderately energy-dense (while being low in sugar content) are ideal early foods for infants [7,8]. The first food exposures should meet the infant’s/toddler’s high nutritional requirements for energy, certain fatty acids, and key vitamins and minerals, such as vitamin A/provitamin A carotenoids, several B vitamins, iodine, iron, and zinc [9,10]. Deficiencies in any of these nutrients during the critical times of development can have both immediate and long-lasting effects on the health of the offspring.

Fruits and vegetables are some of the most consistently recommended early food options, but few of the most popular fruit options such as apples, bananas and grapes have the characteristics of being both moderate in energy-density and low in sugar content. Avocados—technically classified as fleshy, single-seeded berries—differ from most other fruits, however, in that they are a source of several promising non-essential compounds, such as fat-soluble antioxidants and monounsaturated fatty acids (MUFA). The sugar content is less than 1 g per serving, which is the lowest amount compared to all other fresh fruits. A 1-ounce (30 g) serving of avocado is higher in key developmental nutrients such as folate, vitamin E, and lutein compared to a Nutrition Labeling and Education Act (NLEA) serving size of the most popular complementary and transitional feeding fruits (*i.e.*, apples, pears, bananas, grapes, strawberries, and peaches) (Table 1) [11,12,13]. A standard serving of avocados is also less than one fourth the weight of any of the other commonly consumed fruits, making them a very efficient vehicle food for delivering essential nutrition in a portion size that an infant or toddler would more likely be able to consume in one sitting. Avocado is also higher in unsaturated fatty acids compared to most fruits and vegetables [14] (Table 1). The fatty acids in avocados allow for greater absorption of fat-soluble nutrients, either inherent in the fruit or from other foods eaten with avocado, when compared to other fruits and vegetables that are low in fat (or fat-free) [15,16].

This paper is the second of a two-part series on ideal food and nutrient intake and related health outcomes covering the first 1000 days of life (*i.e.*, from the time of conception to the end of a toddler’s second year). The first paper in the series provides evidence for the regular inclusion of key nutrients as well as low-glycemic fruits and vegetables, such as avocados, in a maternal diet for improving birth outcomes and various aspects of maternal health extending from the pre-conceptional period through pregnancy and lactation [17]. This report provides further evidence regarding the regular inclusion of such key nutrients and their food sources, including avocados, on many potential early- and later-life health benefits, as well as explore the potential beneficial effects of monounsaturated fatty acids and bioactive compounds during the complementary and transitional feeding periods. The paper also addresses nutrition topic questions posed by the U.S. Department of Agriculture (USDA) and U.S. Department of Health and Human Services (HHS) expert work groups that comprise the Birth to 24 months and Pregnant Women Dietary Guidance Development Project—specifically Work Group 2–Infancy: Period of Complementary Feeding (6–12 months) and Work Group 3–Period of Transitional Feeding (12–24 months):

- What types and amounts of complementary foods are necessary for infants fed human milk, formula, or mixed feedings to promote favorable health outcomes, such as (1) growth and physical development; (2) cognitive, behavioral or neuromotor development?
- What strategies can be used to improve dietary quality and micronutrient intake in infants six to 12 months of age?
- What are the evidence-based strategies to enhance acceptance of nutrient-dense foods like fruits and vegetables?
- Does exposure (timing, quantity, frequency) to nutrient-dense foods in weaned infants increase acceptance of nutrient-dense foods?
- Does increased acceptance/preference for nutrient-dense foods in the first year of life persist? Does it improve dietary intake of nutrient-dense foods at 12–24 months?
- Does the intake of foods with added salt and sugar in infancy influence the preference and analgesic appeal of dietary salt and sweet in infants, young children, and adults?
- What are the energy requirements for toddlers, ages 12–24 months, to promote optimal growth and physical development?
- What is the relationship between observed intakes of fiber, vitamin A, and folate and the estimated average requirement (EAR) and upper limits (UL) for toddlers 12–24 months of age?
- What food characteristics (e.g., taste/flavor characteristics, portion size, energy, nutrient-density, novel or familiar) impact the development of food preferences and dietary intake?

## 2. Background Information and General Recommendations for Complementary Feeding (6 to 12 Months)

Current guidance from the World Health Organization (WHO) and other leading developed countries is consistent in the recommendations that complementary feedings should begin at about six months of age to support sufficient infant growth and development [19,20,21,22], and should last through the end of the first year of life. During this period breastfeeding should still remain a primary source of nutrition [23]. Therefore, breastfeeding mothers should pay careful attention to the quality of their own diets, since for some nutrients (especially vitamins and fatty acids), the foods mothers eat directly influence the nutrient profile of the milk consumed by their infants [24]. When introducing new foods to an infant, experts recommend that parents and caregivers offer a variety of nutrient-dense, soft- and mixed-textured fruits, vegetables, cereals, and meats that contain little or no added sugar or salt [3,7,25]. The WHO recommends that infants start receiving complementary foods in addition to breast milk two to three times a day from six to eight months of age, and three to four times a day from nine months of age through the end of the complementary feeding period [26].

A consensus statement from the American Heart Association (AHA) on dietary recommendations for children acknowledged that only a small percentage of infants in the U.S. remain exclusively breastfed after four months of age [27]. Corroborating the AHA findings, a recent Hass Avocado Board (HAB) funded survey (HAB Caregiver Survey) conducted among 338 caregivers of infants between the ages of four and 24 months also shows nearly half of the respondents introduced fruits and vegetables between four and six months of age, while approximately one-third introduced protein foods (meat, beans, eggs, peanut butter), grains or dairy in that same time period [28].

Infants will usually triple their body weight between birth and the end of their first year, and much of this growth occurs in the complementary feeding period [29]. In order to accomplish the high rate of growth and development, infants must consume both adequate amounts of energy and essential nutrients. Foods consumed throughout the complementary feeding stage should balance the nutrients from milk or formula, without imposing excess intakes of energy or nutrients [30]. Since complementary foods are initially consumed in very small quantities, caregivers should offer moderately energy-dense foods, rich in multiple nutrients that are key for proper infant health and development (*i.e.*, iron, zinc, calcium, provitamin A/carotenoids, vitamin C, and folate) [8].

## 3. Background Information and General Recommendations for Transitional Feeding (13 to 24 Months)

Similarly to complementary feeding, transitional feeding (13 to 24 months) is defined as all solid and liquid foods consumed other than breast milk and infant formula. The main differences between complementary foods and transitional foods are: (1) the timeframe in which they are consumed (*i.e.*, first year *versus* second year of life); (2) the texture and consistency of foods recommended, which require more developed mastication and digestive abilities; and (3) the portions consumed, based on individual appetite, breast milk and/or formula consumption, and nutritional needs. Toddlers typically consume an increasingly complex diet, moving further away from milk and/or formula towards a diet more focused on a variety of table foods, especially those consumed by other family members [31]. On average, approximately two-thirds of a toddler’s energy intake from 12 to 24 months comes from transitional foods [20,23], more than double the energy intake from table foods when compared to the last months of the complementary period [32].

Currently, little research exists on nutrition and health in the toddler age group (13–24 months). The existing evidence is further complicated by datasets that do not distinguish between age groups and combine one- and two-year-olds together without teasing out the differences between these age groups [1]. This gap in scientific knowledge is reflected in the DRI for toddlers, which were generally derived by extrapolating data from studies involving infants or adults [33]. Toddlers’ nutrition needs are different however. They are less dependent on breast milk for their nutrients and need more fiber, while experiencing greater growth velocity than older children [1]. Therefore ideal toddler foods should contain adequate energy, as well as fiber and key nutrients not found in sufficient amounts in breast milk or other popular family foods [23,34].

## 4. Ideal Complementary and Transitional Foods: Recommendations *versus* Reality

Many of the nutrient-rich foods recommended by the *2015 Dietary Guidelines for Americans* (DGA) [35] for child and adult health have been linked to allergy development and are thought to be contraindicated in the complementary feeding period for infants. These foods include seafood, nuts, and eggs [36,37], although recent evidence has challenged the early exposure hypothesis linking these foods to the development of allergies [38,39]. Other nutrient-dense food options contain too much saturated fat, added salt, sugar, spices, preservatives, additives or artificial ingredients [8,20]. Furthermore, in addition to their nutritional properties, ideal complementary and transitional foods should also have specific physical and chemical attributes such as a proper texture and consistency, along with natural and relatively neutral flavors, to encourage toddlers to develop familiarity and taste preferences for them [20,31].

Data from the landmark Feeding Infants and Toddlers Studies (FITS I and FITS II) along with recent National Health and Nutrition Examination Survey (NHANES) data (from children over two years old) show that young children consume nearly 40% of their total energy from foods like refined grains, sugar-sweetened beverages, and fruit juice. [40,41]. The findings also showed a general decline in fruit and vegetable intake, especially fiber-rich fruits and vegetables as children age from infancy into toddlerhood [27]. The data also showed that infants and toddlers were much more likely to consume sweets, such as cookies or candies rather than nutrient-rich options such as fruits or vegetables [5]. This trend continued through the end of the transitional feeding period, when approximately one-third of U.S. toddlers consumed no fruit on a daily basis [42] and deep yellow vegetable intake went from being consumed by nearly 40% of infants during the middle of the complementary feeding period down to 13% by the time they reached the end of the transitional feeding period [31]. Nutrient- and antioxidant-dense dietary options were consistently replaced by lower-nutrient and higher-energy options such as candies, desserts, and sugar-sweetened beverages [31,43].

The most popular fruits consumed by infants were apples, bananas, peaches, and pears [42], and often times these were in forms that contained added sugars. Apples, bananas, grapes, peaches, and strawberries ranked as the most popular in a child’s second year of life [41,42]. Apples and bananas were also the most popular fruits among infants [28]. In summary, despite the popularity of these fruits, only avocados, actually meet almost all of the expert recommendations (*i.e.*, nutrient-rich, colorful, naturally soft texture, low in sugar/not overly sweet, low-glycemic). This positions avocados as both a unique and ideal food for infants and toddlers.

## 5. Fruits and Vegetables: Early Exposure Can Lead to Life Long Benefits

Fruits and vegetables are vital to a healthy eating pattern as they contain essential nutrients, various forms of fiber, and potentially beneficial bioactive components such as antioxidants and phytosterols [44,45]. Yet, increasing fruit and vegetable exposure and intake among infants/toddlers remains a challenge for both caregivers and dietetic professionals [46,47]. Both fruit and vegetable intake in children of all ages remains below recommendations in most countries worldwide [48], and a large percentage of U.S. infants from six to 12 months of age do not consume any fruits or vegetables on a daily basis [42,49]. A practical goal would be simple dietary changes focused on exchanging empty-calorie foods and beverages for nutrient-rich, high-fiber fruits and vegetables containing no added sugar.

Experts suggest that vegetables and low-sugar fruits, such as avocados, should be introduced in the early stages in order to avoid invoking an early preference for sweet foods, which may influence early childhood and later life eating behaviors [7,8]. The dietary patterns of infants and young children have been shown to correlate to patterns in later childhood [50,51] and even to adulthood [52]; adult health factors, such as cholesterol metabolism, may be programmed from the lipids consumed in infancy [53].

Observational studies have demonstrated later health benefits of healthful early nutrition choices. Better body weights in later years were observed in infants who consumed higher amounts of fruits and/or vegetables and thus less total energy in their diets [5]. Infants given home-prepared fruits or vegetables more frequently at six months of age were more likely to eat more fruits and vegetables several years later compared to infants who were given similar foods less often [54]. Furthermore, frequent fruit and vegetable consumption by younger children was associated with lower blood pressure and a lower risk of stroke in their adult years [55,56], and a lower risk for some cancers [6]. While these studies showed associations between early and later life dietary patterns and health outcomes, very little research has addressed the introduction of specific fruits and vegetables in the complementary and transitional feeding periods, or how consumption of specific “ideal” or popular complementary foods or specific nutrients in those foods (e.g., iron-fortified cereal grains, bananas, apples, avocados, potatoes) may promote long-term health, help build good dietary habits, or assist in reducing health risks. Avocados, and other foods that fit the description of an “ideal complementary food” deserve more clinical research attention, especially since the health status of an infant in its first year of life affects its risk for certain chronic diseases in later years [57].

## 6. Macronutrients: Amount and Specific Structural/Functional Characteristics are Key for Infant and Toddler Health

### 6.1. Dietary Fat: Quantity and Quality Both Matter for Growth and Development

In infancy, fat should comprise about 50% of energy intake in order to provide adequate energy for rapid growth and the essential fatty acids for brain development [58]. With the introduction of complementary foods, energy intake may become inadequate as foods such as fruits, vegetables and cereal grains are generally very low in fat. Complementary foods that are a good source of both fat and energy are important to maintain energy intake. Once in the toddler period, the percentage of fat in the diet may be reduced; however, the total energy and nutrients, including essential fatty acids, must increase to cover the energy cost of activity and growth. Achieving adequate fat intake may be a challenge since in the FITS II data, total fat intakes were below the acceptable macronutrient distribution range in one in four toddlers from the U.S. [59]. Diets of infants and toddlers that are low fat are associated with lower vitamin and mineral intakes [58], and lower fat-soluble vitamin absorption [60]. A Joint Food and Agriculture Organization (FAO)/WHO Expert Consultation report underlined the importance of not only the quantity, but also the quality of fat for proper infant health and development [60].

A joint statement from Health Canada, the Canadian Pediatric Society, Dietitians of Canada, and Breastfeeding Committee for Canada suggests that parents and caregivers should provide adequate amounts of healthy fats in addition to breast milk, and specifically includes avocados as an example of a nutritious fat-containing food for infant health [20]. Avocados are unique among the commonly recommended complementary and transitional fruits and vegetables in that they contain 3.5 g of unsaturated fats per 1-ounce (30 g) serving, accounting for more than 75% of their fat content. The unsaturated fatty acids are primarily in the form of the MUFA oleic acid (C18:1; *n* = 9), while a lesser amount comes from other MUFA, polyunsaturated fatty acids (PUFA), and saturated fatty acids (SFA). Although oleic acid is not considered an essential fatty acid because the human body can synthesize it from other fatty acids, it is the most abundant fatty acid in breast milk [61]. MUFA, such as oleic acid, has also been shown to be important for the normal growth and development of the central nervous system and brain [62], as well as being beneficial for fat-soluble nutrient absorption [15,16]. A 30 g serving of avocado contains approximately 0.5 g of the PUFA linoleic acid (18:2*n*-6) [14], which comprises roughly 10%–15% of the fatty acid content in avocados [63]. Evidence exists showing that as little as 3%–4.5% of total energy intake from linoleic acid is associated with optimal growth and development for infants and toddlers [60].

Since avocados provide energy, MUFA, and PUFA, they would contribute to achieving nutrient balance in infant diets, as well as aiding in absorption of fat-soluble nutrients, providing a source of antioxidants, and thereby potentially contributing to health benefits [16,64,65].

### 6.2. Fiber: Balancing Intake for Optimal Health

Breast milk contains various non-digestible oligosaccharides, which are small-chain prebiotic fiber compounds that are important for infant gut health and immune development [66,67]. While other infant foods are not rich sources of non-digestible oligosaccharides, many fruits and vegetables are rich sources of both soluble and insoluble fibers (e.g., non-digestible carbohydrates and lignin), which may have health benefits even in young children [68]. Currently, there is no infant adequate intake (AI) established for fiber, but it has been suggested by the American Academy of Pediatrics that the amount be gradually increased to provide roughly 5 g per day by the end of the first year of life [69,70].

Neither the appropriate amount nor type of fiber for infants is as yet determined, with both too little and too much dietary fiber posing their own unique set of problems. Too little fiber can lead to constipation [71], and excessive fiber intake has the potential to negatively impact energy and nutrient intake by increasing fecal energy losses, extending satiety (therefore leading to lower energy and nutrient intakes), and binding up minerals through fiber-associated phytates and oxalates [71,72,73]. However, fiber intakes of about 4 g/day in infants at 8 months and 7 g/day in infants at 13 months of age have been positively associated with energy intake and weight gain [74]. Additionally, higher dietary fiber intake in infancy was associated with higher intakes of vitamins and minerals compared to lower fiber intakes [74]. In the FITS I data, infants in the highest quartile of fiber intake were also in the highest quartile of energy and macronutrient intake from table foods, providing further evidence that higher fiber intakes in infancy are not associated with under eating [32].

Concern is expressed by practitioners and researchers about both too much fiber intake in the early feeding periods, and inadequate fiber consumption throughout every life stage thereafter [20,68,69,75]. A decline in fiber intake from the complementary period to transitional period was demonstrated in Finnish infants in which 55%–70% of the fiber in an infant’s diet was from fruits and vegetables, while only 40%–45% of fiber in a toddler’s diet comes from fruits and vegetables [76]. In American infants in FITS I the top sources of dietary fiber for toddlers were primarily refined grains such as non-infant cereals and breads, rolls, biscuits, bagels, and tortillas, along with carbohydrate-rich fruits and vegetables such as bananas and white potatoes [76].

Avocados could contribute to infant fiber intake as they have approximately 2 g of fiber in a 30 g serving [14], which is equal to or greater than nearly all other commonly consumed complementary or transitional foods or fruits by weight [11]. Of the total fiber in avocados, 30%–40% is soluble while 60%–70% is insoluble [14]. When the fiber content of more than 30 fruits and vegetables were compared, avocados stood out as the only food source with relatively high amounts of both soluble fiber (2.1% by weight) and insoluble fiber (2.7% by weight). Additionally, avocados also contain lower levels of phytates and oxalates compared to the most popular fiber sources such as cereal fibers, vegetables, and legumes, thus minimizing loss of calcium and other key essential minerals due to binding by such substances [77].

The higher soluble fiber content of avocados compared to other fruits may be of benefit to the development of an infant’s/toddler’s gut microflora as it is fermented by the colonic microflora to a greater extent. While fermentable soluble fibers (*i.e.*, prebiotics such as oligosaccharides) from breast milk are known to have potent beneficial effects on infant health [78], the dose response and potential effects of plant-based soluble fiber sources on infant health requires further research. The soluble fiber in avocados may also contribute an energy source for infants since the fermentable nature of soluble fiber allows the colonic microflora to metabolize undigested polysaccharides and produce various short-chain fatty acids (e.g., acetic acid, butyric acid and propionic acid), which are then able to be absorbed [79,80,81].

## 7. Micronutrients: Avoiding Deficiencies during the Complementary and Transitional Feeding Periods

### 7.1. Nutrients for Building the Blood

The selection of complementary foods to meet the micronutrient needs of infants is challenging as essential nutrients like iron are present in low concentrations in typical infant foods, even in breast milk [82]. Avocados, while low in iron, contain folate, vitamin C, riboflavin, and vitamin B6 that are all essential to various aspects of iron absorption, red blood cell formation and/or hemoglobin function. Vitamin C enhances non-heme iron absorption and is a key factor in its bioavailability [83,84,85]. Folate is critical for the proper synthesis of red blood cells; and is therefore important for prevention of megaloblastic anemia [86]. Vitamin B6 plays a role in the synthesis of hemoglobin and in oxygen transport, and a deficiency in vitamin B6 can lead to microcytic hypochromic anemia. Riboflavin is required for the enzymatic activation of folate and vitamin B6 as well as for red blood cell production, and a deficiency in riboflavin can lead to normocytic anemia. Foods such as avocados, should be considered as providing a unique combination of several blood-building nutrients that can act as iron-absorption enhancers and/or function in red blood cell synthesis.

### 7.2. Potassium

As infants and toddlers begin to consume less electrolyte-rich breast milk, complementary food sources must provide a balance of electrolytes necessary to maintain proper fluid balance and bone turnover. However, it is observed that about 45%–80% of toddlers exceed the recommended sodium intake levels, and only 5% of toddlers meet the recommended intake levels for potassium [59,87]. To reduce sodium and provide potassium, complementary foods like avocados offer not only a sodium-free, complementary food, but they are also rich in potassium (Table 1) [14].

### 7.3. Enhanced Fat-Soluble Nutrient Absorption

Natural food sources of lipid-soluble vitamins and antioxidant compounds are important to identify as it is known that toddlers receive a substantial amount of nutrients, such as vitamin A, from supplements and fortified foods instead of from unprocessed whole foods [76]. More than 60% of toddlers in the U.S. consume less vitamin E than the EAR [59]. Avocados contribute three of the four fat-soluble vitamins such that a 1-ounce serving of avocado provides more provitamin A in the form of carotenoids than almost all other fruits, as well as small amounts of vitamin E and vitamin K (Table 1). Absorption of fat-soluble vitamins is enhanced in the presence of adequate fat intake, which is known to be less than adequate in the toddler age group [58]. The MUFA content of avocados is unique among other fruits and vegetables as fatty acids help fat-soluble vitamins and carotenoids (e.g., lutein, lycopene, alpha-carotene, and beta-carotene) be more effectively absorbed from other foods [15,16]. Avocado consumption can also enhance the efficiency of conversion of carotenoids to vitamin A by two to six fold [16].

## 8. Avocado Dietary Bioactive Components: Playing an Important Role in Infant Health

Breast milk is known to have numerous bioactive properties (*i.e.*, properties above and beyond their nutritive roles) that are associated with infant health and development [88,89,90]. Complementary and transitional foods contain bioactive components such as fiber, antioxidants, electrolytes, carotenoids, and flavonoids [14,91]. Avocados also contain many lipophilic phytochemicals and bioactive compounds that may confer health benefits (e.g., sterols, polyhydroxylated fatty alcohols (PFA), alkaloids, acetogenins, and volatile oils) [91,92,93]. While understanding of the interplay of antioxidant, prebiotic and sterol components in foods and food combinations is just emerging, it has been suggested that a wide variety of bioactive compounds are responsible for the health benefits of fruits and vegetables through additive and synergistic interactions by targeting multiple signal transduction pathways [94].

For early infant foods, such as fruits and vegetables, the health effects are attributed to different bioactive compounds such as vitamin C, carotenoids, and various phenolic compounds [95]. The antioxidant potential of components like carotenoids (beta-carotene, lutein and zeaxanthin) in fruits and vegetables not only provide the precursors for vitamin A (which is essential for proper growth, development, vision, immunity, hair and skin health, and mucous membrane formation) [25], but may also act as free-radical scavenging antioxidants [96]. Additionally, these carotenoids have functional roles in the tissues of the infant brain [97].

Lutein accounts for the majority of infant brain carotenoids, representing approximately 60% of total carotenoids [98]. Recent metabolomics studies on post-mortem infants showed correlations between brain lutein concentrations and energy metabolite pathways, lipid metabolite pathways, and amino acid neurotransmission pathways [99]. Further, formula-fed infants compared to breast-fed infants had significantly lower lutein concentrations in their blood [100]. Lutein was approved by the FDA for use in infant formulas, despite it not being officially classified as an essential nutrient. As complementary foods begin to displace breast milk or formula in the diet, adequate sources of lutein from complementary foods may be important to infant health [99]. Avocados contain some of the highest levels of lutein and dietary fat of any fruit or vegetable (Table 1), along with the added benefit of a dietary MUFA fat source to aid absorption of the fat-soluble lutein [15,16].

Phytosterols and PFA are two other lipid-soluble compounds that account for a large portion of the lipid content of avocados but their potential benefits have not been studied in infants or toddlers [25]. In adults, both phytosterols and PFA have been shown to support a healthy inflammatory response [25]. Lipophilic acetogenins in avocados [92] are a group of antioxidants that are synthesized from fatty acid precursors that have promise for their anti-proliferative and apoptotic effects on cancer cells [101,102,103]. The amino-acid based antioxidant glutathione, also in avocados in higher concentration (8.4 mg/30 g) that any other fruit [104], is involved in immune function, lipid metabolism, detoxification, and several aspects of cellular defense and replication [105]. Since heating and processing reduces glutathione levels in foods, foods which are commonly consumed raw, such as avocados, contain higher levels of this antioxidant compound. Future research is required to identify the types of foods with fat-soluble nutrient absorption-enhancing properties that may optimize infant and toddler health, as well as provide protection against free-radical damage and future chronic disease risk [95].

## 9. Food Preferences: Early Exposure to Flavor and Texture Can Influence Acceptability

Beyond choosing the most ideal nutrient-rich foods to feed their infants and toddlers, parents and caregivers should also understand the roles that the flavors and textures of foods play in transitional and complementary feeding. Food learning and flavor preferences start in utero and are heavily influenced by breastfeeding and the infant’s complementary diet in the first year of life [106]. The early taste preferences appear to be biologically driven with certain flavors, such as sweetness indicating available calories, and bitter flavors indicating potentially dangerous compounds present in the food [106]. These preferences have been shown to be somewhat malleable and dependent on environmental factors such as repeated exposures to flavors [1,107]. Once established, many of the early dietary preferences and habits tend to have a long-lasting influence [47], even into adulthood where there is a tendency to favor foods the way they were initially introduced [8]. Therefore, proper early food exposure is important for laying the foundation of a long-term varied diet. Beyond taste and texture, early exposure to a myriad of food taste and texture profiles also teaches societal and familial ideals, attitudes, and beliefs about food and eating behaviors [108].

In order to establish a varied eating pattern—which includes neutral, sour, and bitter taste acceptance—the ideal initial foods should be those that are both nutritious and have a low to moderate sweet and salty flavor profile [8]. Such presentation takes advantage of the plasticity of early flavor learning [106]. Additionally, infants who have positive early experiences with fruits and vegetables are significantly more likely to choose and consume those foods later in life [109,110,111]. Unfortunately, findings from the HAB Caregiver Survey indicate that this approach is not being readily followed. Nearly 60% of infants and toddlers were described as picky eaters, and the most common foods offered and consumed early in life were sweet fruits and starchy vegetables [28]. Further, the caregivers’ overarching goal was to give “foods that the infant/toddler really enjoyed eating”, while the actual nutritional value of the food provided ranked lower on their agendas [28]. In order to combat the overly sweet and salty flavors of the standard American diet without offending the child’s innate dislikes for bitter and sour, some pediatricians recommend introducing mild foods (*i.e.*, neither sweet, salty, sour, or bitter) with a neutral flavor profile in the early complementary period [7]. Avocados can provide children with the types of nutrients and phytochemicals found in many sweet-tasting, sugar-rich fruits and bitter-tasting vegetables.

In addition to flavor preferences, infants also have texture preferences due to their developing abilities to chew and swallow. When foods are being introduced to infants it is important that caregivers provide a variety of soft textures—such as creamy, lumpy, tender, pureed, mashed or ground—in order to properly develop oro-sensory functions and the swallowing mechanism [20]. Different textures are necessary to gradually introduce the baby to solid foods while reducing the risk of choking or swallowing large chunks of food that are difficult to digest. Soft fruit and vegetable consumption—such as from peaches, bananas, and avocados—is consistent with several recommendations from the federal feeding assistance program Special Supplemental Nutrition Program for Women, Infants, and Children (WIC) for infant feeding (although peaches and bananas do not have an ideal sweetness/sugar factor). Soft, neutral-flavored, and nutrient-dense avocado—which does not need to be cooked and can easily be stored—appears to be one of the most ideal complementary and transitional foods available. In essence, the avocado’s natural characteristics match what health professionals and caregivers are most likely to consider important for an infant’s first food offerings [28].

## 10. Future Guidelines for Complementary and Transitional Feeding: Importance of Clear and Specific Recommendations

A consistent message from recent literature is that fruit and vegetable intake needs to be increased in infant and toddler dietary patterns; selection of those foods that are lower in sugar and higher in fiber should be recommended above varieties that are higher in sugar and overly sweet. Most specific recommendations for parents and caregivers suggest offering a wide variety of fruits and vegetables on a daily basis, with an emphasis on colorful fruits and dark green, leafy, and deep yellow vegetables [31]. For fruit, recommendations could be clearer to ensure caregivers are making the best choices by specifically calling out: “colorful fruits that are a good source of fiber and low in sugar”. Further, naming of specific examples of these types of fruits would limit confusion and clearly point caregivers to the best options for infants and toddlers.

Avocados are a good example of a fruit that could be specifically recommended as an optimal transitional food. Beyond its texture, flavor and nutrient profile, avocado consumption among infants and toddlers may be able to displace empty calorie offerings more effectively than other nutrient-rich complementary and transitional foods due to their higher amount of appetite suppressing fatty acids and fiber [112,113]. According to the American Dietetic Association (now the Academy of Nutrition and Dietetics), “foods that are rich in energy and nutrients such as avocado should be used when the infant is being weaned [34]”. Therefore, the avocado with its fiber-content, MUFA, moderate energy-density, more than 20 vitamins and minerals, and array of phytonutrients appears to one of the most ideal fruits—and possibly foods—for complementary and transitional feeding [114].

## 11. Conclusions

Major transitions occur in the dietary patterns of infants and toddlers over the first two years of life. Exposure to certain foods and nutrients during the first two years may impact their future health through metabolic programming or development of specific tastes [115]. The most ideal complementary and transitional foods—nutritionally and physiochemically—should be offered regularly to infants and toddlers in order to ensure their optimal health, as well to expand their range of flavor preferences and acceptance for nutrient-rich dietary options. As detailed in this paper, avocados are unique among complementary and transitional foods in that they:

- Contain a spectrum of essential and non-essential nutrients with potential health benefits that minimize undesirable components such as sodium, empty calories, and unhealthy fats.
- Provide an ideal source of energy (high in healthy unsaturated fats and low in sugar) to meet the increasing energy and growth demands of weaning infants and toddlers.
- By weight and serving size, contain some of the highest levels of the antioxidants lutein, zeaxanthin, and glutathione among complementary and transitional foods.
- Are rich in unsaturated fatty acids, which significantly enhance the absorption of lipid-soluble compounds.
- Contain more total fiber and soluble fiber per gram than almost all other complementary and transitional foods, and at the same time contain less mineral-binding phytates and oxalates than other popular high-fiber foods.
- Have a neutral flavor and smooth consistency that is ideal for early infant foods.

At present, the current infant feeding recommendations tend to be based on anecdotal and observational findings, which are largely dependent on an infant’s learned preference for sweet foods. Future development of complementary and transitional feeding recommendations should utilize evidence from controlled studies that investigate the critical nutrient needs of infants and toddlers, and should progress toward identifying the most ideal foods (*i.e.*, those that meet the majority of recommended guidelines). Future research on ideal infant and toddler foods, including avocados, is warranted to further explore their potential in both early life and later life health outcomes.

## Figures and Tables

**Table 1:** Meeting the developmental needs of infants and toddlers. Comparison of a serving (30 g) of avocado *versus* a Nutrition Labeling and Education Act (NLEA) serving (Range: 126–242 g) of the most popular complementary and transitional fruits.

Per NLEA Serving	Apples (242 g)	Avocados (30 g)	Bananas (126 g)	Grapes (126 g)	Peaches (147 g)	Pears (166 g)	Strawberries (147 g)
**>150 mg potassium/serving**	√	√	√	√	√	√	√
**>25 μg folate/serving**		√	√				√
**>0.50 mg α-tocopherol/serving**		√			√		
**>80 μg Lutein + zeaxanthin/serving**		√		√	√		
**>40 IU vitamin A/serving**	√	√	√	√	√	√	
**>6 μg vitamin K/serving**		√		√		√	
**>2.5 g MUFA/serving**		√					
**≥2 g fiber/serving**	√	√	√		√	√	√

Data sourced from: USDA Agricultural Research Service, National Nutrient Database for Standard Reference Release 27 [18]. Basic Report: 09003, apples, raw, with skin; 09038, avocados, raw, California; 09040, bananas, raw; 09131, grapes, American type (slip skin) raw; 09236, peaches, yellow, raw; 09252, pears, raw; 09316, strawberries, raw.

## References

[1] Birch L.L., Doub A.E. (2014). Learning to eat: Birth to age 2 years. Am. J. Clin. Nutr..

[2] Solomons N.W., Vossenaar M. (2013). Nutrient Density in Complementary Feeding of Infants and Toddlers. Eur. J. Clin. Nutr..

[3] USDA (2002). Food and Nutrition Service: Feeding Infants: A Guide for Use in Child Nutrition Programs.

[4] Gale C.R., Martyn C.N., Marriott L.D., Limond J., Crozier S., Inskip H.M., Godfrey K.M., Law C.M., Cooper C., Robinson S.M. (2009). Dietary Patterns in Infancy and Cognitive and Neuropsychological Function in Childhood. J. Child Psychol. Psychiatry.

[5] Saavedra J.M., Deming D., Dattilo A., Reidy K. (2013). Lessons from the Feeding Infants and Toddlers Study in North America: What Children Eat, and Implications for Obesity Prevention. Ann. Nutr. Metab..

[6] Maynard M., Gunnell D., Emmett P., Frankel S., Davey Smith G. (2003). Fruit, Vegetables, and Antioxidants in Childhood and Risk of Adult Cancer: The Boyd Orr Cohort. J. Epidemiol. Community Health.

[7] Stettler N., Bhatia J., Parish A., Stallings V.A., Kliegman R.M., Stanton B.M.D., Geme J.S., Schor N.F., Behrman R.E. (2011). Feeding Healthy Infants, Children, and Adolescents. Nelson Textbook of Pediatrics.

[8] Monte C.M., Giugliani E.R. (2004). Recommendations for the Complementary Feeding of the Breastfed Child. J. Pediatr. (Rio J.).

[9] Ramakrishnan U., Grant F., Goldenberg T., Zongrone A., Martorell R. (2012). Effect of Women’s Nutrition before and During Early Pregnancy on Maternal and Infant Outcomes: A Systematic Review. Paediatr. Perinat. Epidemiol..

[10] Cetin I., Berti C., Calabrese S. (2010). Role of Micronutrients in the Periconceptional Period. Hum. Reprod. Update.

[11] USDA (2014). National Nutrient Database for Standard Reference Release 27: Fiber, Total Dietary (G): “Compared to Fruits and Fruit Juices”. https://ndb.nal.usda.gov/.

[12] USDA (2014). National Nutrient Database for Standard Reference Release 27: Sugars, Total (G): “Compared to Fruits and Fruit Juices”. https://ndb.nal.usda.gov/.

[13] USDA (2014). National Nutrient Database for Standard Reference Release 27: Fatty Acids, Total Monounsaturated (G): “Compared to Fruits and Fruit Juices”. https://ndb.nal.usda.gov/.

[14] Dreher M.L., Davenport A.J. (2013). Hass Avocado Composition and Potential Health Effects. Crit. Rev. Food Sci. Nutr..

[15] Unlu N.Z., Bohn T., Clinton S.K., Schwartz S.J. (2005). Carotenoid Absorption from Salad and Salsa by Humans Is Enhanced by the Addition of Avocado or Avocado Oil. J. Nutr..

[16] Kopec R.E., Cooperstone J.L., Schweiggert R.M., Young G.S., Harrison E.H., Francis D.M., Clinton S.K., Schwartz S.J. (2014). Avocado Consumption Enhances Human Postprandial Provitamin a Absorption and Conversion from a Novel High-Beta-Carotene Tomato Sauce and from Carrots. J. Nutr..

[17] Comerford K.B., Ayoob K.T., Murray R.D., Atkinson S.A. (2016). The Role of Avocados in Maternal Diets During the Periconceptional Period, Pregnancy, and Lactation. Nutrients.

[18] US Department of Agriculture, Agricultural Research Service, Nutrient Data Laboratory (2014). USDA National Nutrient Database for Standard Reference, Release 27. http://www.ars.usda.gov/nea/bhnrc/ndl.

[19] Michaelsen K.F., Larnkjaer A., Lauritzen L., Molgaard C. (2010). Science Base of Complementary Feeding Practice in Infancy. Curr. Opin. Clin. Nutr. Metab. Care.

[20] Health Canada: Infant Feeding Joint Working Group Nutrition for Healthy Term Infants: Recommendations from Six to 24 months, 2014. http://www.Hc-Sc.Gc.Ca/Fn-an/Nutrition/Infant-Nourisson/Recom/Recom-6-24-Months-6-24-Mois-Eng.Php#A3.

[21] Australia National Health and Medical Research Council (2012). Infant Feeding Guidelines Information for Health Workers. http://www.Eatforhealth.Gov.Au/Sites/Default/Files/Files/the_Guidelines/N56_Infant_Feeding_Guidelines.Pdf.

[22] (2015). National Health Service: Pregnancy and Baby. http://www.Nhs.Uk/Conditions/Pregnancy-and-Baby/Pages/Solid-Foods-Weaning.Aspx#Close.

[23] WHO (2009). Infant and Young Child Feeding: Model Chapter for Textbooks for Medical Students and Allied Health Professionals.

[24] Champ M., Hoebler C. (2009). Functional Food for Pregnant, Lactating Women and in Perinatal Nutrition: A Role for Dietary Fibres?. Curr. Opin. Clin. Nutr. Metab. Care.

[25] USDA (2009). Food and Nutrition Service: Woman, Infants and Children (Wic). http://www.Nal.Usda.Gov/Wicworks/Topics/Fg/Completeifg.Pdf.

[26] WHO (2014). Nutrition: Complementary Feeding. http://www.Who.Int/Nutrition/Topics/Complementary_Feeding/En/.

[27] Gidding S.S., Dennison B.A., Birch L.L., Daniels S.R., Gillman M.W., Lichtenstein A.H., Rattay K.T., Steinberger J., Stettler N., Van Horn L. (2005). Dietary Recommendations for Children and Adolescents: A Guide for Practitioners: Consensus Statement from the American Heart Association. Circulation.

[28] (2014). Hass Avocado Board: Infant & Toddler Caregiver Survey. http://www.Hassavocadoboard.Com.

[29] Kathy C. (2010). Complementary Feeding for Infants 6 to 12 months. J. Fam. Health Care.

[30] Kuo A.A., Inkelas M., Slusser W.M., Maidenberg M., Halfon N. (2011). Introduction of Solid Food to Young Infants. Mater. Child Health J..

[31] Fox M.K., Pac S., Devaney B., Jankowski L. (2004). Feeding Infants and Toddlers Study: What Foods Are Infants and Toddlers Eating?. J. Am. Diet. Assoc..

[32] Briefel R.R., Reidy K., Karwe V., Jankowski L., Hendricks K. (2004). Toddlers’ Transition to Table Foods: Impact on Nutrient Intakes and Food Patterns. J. Am. Diet. Assoc..

[33] Otten J.J., Hellwig J.P., Meyers L.D., Medicine I.O. (2006). Dietary Reference Intakes: The Essential Guide to Nutrient Requirements.

[34] Craig W.J., Mangels A.R., American Dietetic A. (2009). Position of the American Dietetic Association: Vegetarian Diets. J. Am. Diet. Assoc..

[35] U.S. Department of Health, Human Services, U.S. Department of Agriculture 2015–2020 Dietary Guidelines for Americans, 8th ed. http://www.cnpp.usda.gov/2015-2020-dietary-guidelines-americans.

[36] Fiocchi A., Assa’ad A., Bahna S. (2006). Food Allergy and the Introduction of Solid Foods to Infants: A Consensus Document. Adverse Reactions to Foods Committee, American College of Allergy, Asthma and Immunology. Ann. Allergy Asthma Immunol..

[37] Chin B., Chan E.S., Goldman R.D. (2014). Early Exposure to Food and Food Allergy in Children. Can. Fam. Phys..

[38] Pham-Thi N., Bidat E. (2014). Solid Food Introduction and Allergic Risk. Arch. Pediatr..

[39] Abrams E.M., Becker A.B. (2015). Food Introduction and Allergy Prevention in Infants. CMAJ.

[40] Reedy J., Krebs-Smith S.M. (2010). Dietary Sources of Energy, Solid Fats, and Added Sugars among Children and Adolescents in the United States. J. Am. Diet. Assoc..

[41] Fox M.K., Condon E., Briefel R.R., Reidy K.C., Deming D.M. (2010). Food Consumption Patterns of Young Preschoolers: Are They Starting off on the Right Path?. J. Am. Diet. Assoc..

[42] Siega-Riz A.M., Deming D.M., Reidy K.C., Fox M.K., Condon E., Briefel R.R. (2010). Food Consumption Patterns of Infants and Toddlers: Where Are We Now?. J. Am. Diet. Assoc..

[43] Mennella J.A., Ziegler P., Briefel R., Novak T. (2006). Feeding Infants and Toddlers Study: The Types of Foods Fed to Hispanic Infants and Toddlers. J. Am. Diet. Assoc..

[44] Slavin J.L., Lloyd B. (2012). Health Benefits of Fruits and Vegetables. Adv. Nutr..

[45] Poiroux-Gonord F., Bidel L.P., Fanciullino A.L., Gautier H., Lauri-Lopez F., Urban L. (2010). Health Benefits of Vitamins and Secondary Metabolites of Fruits and Vegetables and Prospects to Increase Their Concentrations by Agronomic Approaches. J. Agric. Food Chem..

[46] Ashman A.M., Collins C.E., Hure A.J., Jensen M., Oldmeadow C. (2014). Maternal Diet During Early Childhood, but Not Pregnancy, Predicts Diet Quality and Fruit and Vegetable Acceptance in Offspring. Mater. Child Nutr..

[47] Hetherington M.M., Schwartz C., Madrelle J., Croden F., Nekitsing C., Vereijken C.M., Weenen H. (2015). A Step-by-Step Introduction to Vegetables at the Beginning of Complementary Feeding. The Effects of Early and Repeated Exposure. Appetite.

[48] de Lauzon-Guillain B., Jones L., Oliveira A., Moschonis G., Betoko A., Lopes C., Moreira P., Manios Y., Papadopoulos N.G., Emmett P. (2013). The Influence of Early Feeding Practices on Fruit and Vegetable Intake among Preschool Children in 4 European Birth Cohorts. Am. J. Clin. Nutr..

[49] Young B.E., Krebs N.F. (2013). Complementary Feeding: Critical Considerations to Optimize Growth, Nutrition, and Feeding Behavior. Curr. Pediatr. Rep..

[50] Northstone K., Emmett P.M. (2008). Are Dietary Patterns Stable Throughout Early and Mid-Childhood? A Birth Cohort Study. Br. J. Nutr..

[51] Golley R.K., Smithers L.G., Mittinty M.N., Emmett P., Northstone K., Lynch J.W. (2013). Diet Quality of U.K. Infants Is Associated with Dietary, Adiposity, Cardiovascular, and Cognitive Outcomes Measured at 7–8 years of Age. J. Nutr..

[52] Mikkila V., Rasanen L., Raitakari O.T., Pietinen P., Viikari J. (2005). Consistent Dietary Patterns Identified from Childhood to Adulthood: The Cardiovascular Risk in Young Finns Study. Br. J. Nutr..

[53] Owen C.G., Whincup P.H., Kaye S.J., Martin R.M., Davey Smith G., Cook D.G., Bergstrom E., Black S., Wadsworth M.E., Fall C.H. (2008). Does Initial Breastfeeding Lead to Lower Blood Cholesterol in Adult Life? A Quantitative Review of the Evidence. Am. J. Clin. Nutr..

[54] Coulthard H., Harris G., Emmett P. (2010). Long-Term Consequences of Early Fruit and Vegetable Feeding Practices in the United Kingdom. Public Health Nutr..

[55] Moore L.L., Singer M.R., Bradlee M.L., Djousse L., Proctor M.H., Cupples L.A., Ellison R.C. (2005). Intake of Fruits, Vegetables, and Dairy Products in Early Childhood and Subsequent Blood Pressure Change. Epidemiology.

[56] Ness A.R., Maynard M., Frankel S., Smith G.D., Frobisher C., Leary S.D., Emmett P.M., Gunnell D. (2005). Diet in Childhood and Adult Cardiovascular and All Cause Mortality: The Boyd Orr Cohort. Heart.

[57] U.S. Department of Agriculture, U.S. Department of Health and Human Services (2010). Dietary Guidelines for Americans.

[58] Picciano M.F., Smiciklas-Wright H., Birch L.L., Mitchell D.C., Murray-Kolb L., McConahy K.L. (2000). Nutritional Guidance Is Needed During Dietary Transition in Early Childhood. Pediatrics.

[59] Butte N.F., Fox M.K., Briefel R.R., Siega-Riz A.M., Dwyer J.T., Deming D.M., Reidy K.C. (2010). Nutrient Intakes of Us Infants, Toddlers, and Preschoolers Meet or Exceed Dietary Reference Intakes. J. Am. Diet. Assoc..

[60] Fats and Fatty Acids in Human Nutrition (2009). Proceedings of the Joint Fao/Who Expert Consultation. 10–14 November 2008. Geneva, Switzerland. Ann. Nutr. Metab..

[61] Saphier O., Blumenfeld J., Silberstein T., Tzor T., Burg A. (2013). Fatty Acid Composition of Breastmilk of Israeli Mothers. Indian Pediatr..

[62] Martysiak-Zurowska D., Zoralska K., Zagierski M., Szlagtys-Sidorkiewicz A. (2011). Fatty Acid Composition in Breast Milk of Women from Gdansk and the Surrounding District in the Course of Lactation. Med. Wieku Rozwoj..

[63] Lu Q.Y., Zhang Y., Wang Y., Wang D., Lee R.P., Gao K., Byrns R., Heber D. (2009). California Hass Avocado: Profiling of Carotenoids, Tocopherol, Fatty Acid, and Fat Content During Maturation and from Different Growing Areas. J. Agric. Food Chem..

[64] Stunkard A.J., Berkowitz R.I., Schoeller D., Maislin G., Stallings V.A. (2004). Predictors of Body Size in the First 2 years of Life: A High-Risk Study of Human Obesity. Int. J. Obes. Relat. Metab. Disord..

[65] Heppe D.H., Kiefte-de Jong J.C., Durmus B., Moll H.A., Raat H., Hofman A., Jaddoe V.W. (2013). Parental, Fetal, and Infant Risk Factors for Preschool Overweight: The Generation R Study. Pediatr. Res..

[66] Kapiki A., Costalos C., Oikonomidou C., Triantafyllidou A., Loukatou E., Pertrohilou V. (2007). The Effect of a Fructo-Oligosaccharide Supplemented Formula on Gut Flora of Preterm Infants. Early Hum. Dev..

[67] Euler A.R., Mitchell D.K., Kline R., Pickering L.K. (2005). Prebiotic Effect of Fructo-Oligosaccharide Supplemented Term Infant Formula at Two Concentrations Compared with Unsupplemented Formula and Human Milk. J. Pediatr. Gastroenterol. Nutr..

[68] Kranz S., Brauchla M., Slavin J.L., Miller K.B. (2012). What Do We Know About Dietary Fiber Intake in Children and Health? The Effects of Fiber Intake on Constipation, Obesity, and Diabetes in Children. Adv. Nutr..

[69] Agostoni C., Riva E., Giovannini M. (1995). Dietary Fiber in Weaning Foods of Young Children. Pediatrics.

[70] Committee on Nutrition, American Academy of Pediatrics (1998). Pediatric Nutrition Handbook.

[71] Stewart M.L., Schroeder N.M. (2013). Dietary Treatments for Childhood Constipation: Efficacy of Dietary Fiber and Whole Grains. Nutr. Rev..

[72] Hambidge K.M. (2010). Micronutrient Bioavailability: Dietary Reference Intakes and a Future Perspective. Am. J. Clin. Nutr..

[73] Moilanen B.C. (2004). Vegan Diets in Infants, Children, and Adolescents. Pediatr. Rev..

[74] Ruottinen S., Lagstrom H.K., Niinikoski H., Ronnemaa T., Saarinen M., Pahkala K.A., Hakanen M., Viikari J.S., Simell O. (2010). Dietary Fiber Does Not Displace Energy but Is Associated with Decreased Serum Cholesterol Concentrations in Healthy Children. Am. J. Clin. Nutr..

[75] Donini L.M., Savina C., Cannella C. (2009). Nutrition in the Elderly: Role of Fiber. Arch. Gerontol. Geriatr..

[76] Fox M.K., Reidy K., Novak T., Ziegler P. (2006). Sources of Energy and Nutrients in the Diets of Infants and Toddlers. J. Am. Diet. Assoc..

[77] Kasidas G.P., Rose G.A. (1980). Oxalate Content of Some Common Foods: Determination by an Enzymatic Method. J. Hum. Nutr..

[78] Bode L. (2012). Human Milk Oligosaccharides: Every Baby Needs a Sugar Mama. Glycobiology.

[79] Cummings J.H., Englyst H.N. (1987). Fermentation in the Human Large Intestine and the Available Substrates. Am. J. Clin. Nutr..

[80] Kaji I., Karaki S., Kuwahara A. (2014). Short-Chain Fatty Acid Receptor and Its Contribution to Glucagon-Like Peptide-1 Release. Digestion.

[81] den Besten G., van Eunen K., Groen A.K., Venema K., Reijngoud D.J., Bakker B.M. (2013). The Role of Short-Chain Fatty Acids in the Interplay between Diet, Gut Microbiota, and Host Energy Metabolism. J. Lipid Res..

[82] Dewey K.G. (2013). The Challenge of Meeting Nutrient Needs of Infants and Young Children During the Period of Complementary Feeding: An Evolutionary Perspective. J. Nutr..

[83] Vitolo M.R., Bortolini G.A. (2007). Iron Bioavailability as a Protective Factor against Anemia among Children Aged 12 to 16 months. J. Pediatr. (Rio J.).

[84] Hazell T. (1987). Vitamin C Has a Key Physiological Role in Facilitating the Absorption of Non-Heme Iron from the Diet. Hum. Nutr. Appl. Nutr..

[85] Cook J.D., Reddy M.B. (2001). Effect of Ascorbic Acid Intake on Nonheme-Iron Absorption from a Complete Diet. Am. J. Clin. Nutr..

[86] Pacheco M.M., Gomez R.L., Garciglia R.S., Calderon M.R., Muñoz R.E.M. (2011). Folates and *Persea americana* Mill (Avocado). Emir. J. Food Agric..

[87] Tian N., Zhang Z., Loustalot F., Yang Q., Cogswell M.E. (2013). Sodium and Potassium Intakes among US Infants and Preschool Children, 2003–2010. Am. J. Clin. Nutr..

[88] Casazza K., Hanks L.J., Fields D.A. (2014). The Relationship between Bioactive Components in Breast Milk and Bone Mass in Infants. Bonekey Rep..

[89] Lonnerdal B. (2013). Bioactive Proteins in Breast Milk. J. Paediatr. Child Health.

[90] McEvoy B. (1984). Transfer of Bioactive Substances in Breast Milk. Med. J. Aust..

[91] Yasir M., Das S., Kharya M.D. (2010). The Phytochemical and Pharmacological Profile of Persea Americana Mill. Pharmacogn. Rev..

[92] Rodriguez-Sanchez D., Silva-Platas C., Rojo R.P., Garcia N., Cisneros-Zevallos L., Garcia-Rivas G., Hernandez-Brenes C. (2013). Activity-Guided Identification of Acetogenins as Novel Lipophilic Antioxidants Present in Avocado Pulp (*Persea Americana*). J. Chromatogr. B Anal. Technol. Biomed. Life Sci..

[93] Rodriguez-Sanchez D.G., Flores-Garcia M., Silva-Platas C., Rizzo S., Torre-Amione G., De la Pena-Diaz A., Hernandez-Brenes C., Garcia-Rivas G. (2014). Isolation and Chemical Identification of Lipid Derivatives from Avocado (Persea Americana) Pulp with Antiplatelet and Antithrombotic Activities. Food Funct..

[94] Liu R.H. (2013). Dietary Bioactive Compounds and Their Health Implications. J. Food Sci..

[95] Carbonell-Capella J.M., Barba F.J., Esteve M.J., Frigola A. (2014). Quality Parameters, Bioactive Compounds and Their Correlation with Antioxidant Capacity of Commercial Fruit-Based Baby Foods. Food Sci. Technol. Int..

[96] Lee J., Koo N., Min D.B. (2004). Reactive Oxygen Species, Aging, and Antioxidative Nutraceuticals. Compr. Rev. Food Sci. Food Saf..

[97] Moukarzel A.A., Bejjani R.A., Fares F.N. (2009). Xanthophylls and Eye Health of Infants and Adults. J. Med. Liban..

[98] Vishwanathan R., Kuchan M.J., Sen S., Johnson E.J. (2014). Lutein and Preterm Infants with Decreased Concentrations of Brain Carotenoids. J. Pediatr. Gastroenterol. Nutr..

[99] Lieblein-Boff J.C., Johnson E.J., Kennedy A.D., Lai C.S., Kuchan M.J. (2015). Exploratory Metabolomic Analyses Reveal Compounds Correlated with Lutein Concentration in Frontal Cortex, Hippocampus, and Occipital Cortex of Human Infant Brain. PLoS ONE.

[100] Bettler J., Zimmer J.P., Neuringer M., DeRusso P.A. (2010). Serum Lutein Concentrations in Healthy Term Infants Fed Human Milk or Infant Formula with Lutein. Eur. J. Nutr..

[101] Silva-Platas C., Garcia N., Fernandez-Sada E., Davila D., Hernandez-Brenes C., Rodriguez D., Garcia-Rivas G. (2012). Cardiotoxicity of Acetogenins from Persea Americana Occurs through the Mitochondrial Permeability Transition Pore and Caspase-Dependent Apoptosis Pathways. J. Bioenerg. Biomembr..

[102] Kojima N., Fushimi T., Tatsukawa T., Yoshimitsu T., Tanaka T., Yamori T., Dan S., Iwasaki H., Yamashita M. (2013). Structure-Activity Relationships of Hybrid Annonaceous Acetogenins: Powerful Growth Inhibitory Effects of Their Connecting Groups between Heterocycle and Hydrophobic Carbon Chain Bearing Thf Ring on Human Cancer Cell Lines. Eur. J. Med. Chem..

[103] Matsui Y., Takeuchi T., Kumamoto-Yonezawa Y., Takemura M., Sugawara F., Yoshida H., Mizushina Y. (2010). The Relationship between the Molecular Structure of Natural Acetogenins and Their Inhibitory Activities Which Affect DNA Polymerase, DNA Topoisomerase and Human Cancer Cell Growth. Exp. Ther. Med..

[104] Jones D.P., Coates R.J., Flagg E.W., Eley J.W., Block G., Greenberg R.S., Gunter E.W., Jackson B. (1992). Glutathione in Foods Listed in the National Cancer Institute’s Health Habits and History Food Frequency Questionnaire. Nutr. Cancer.

[105] Michel S.H., Maqbool A., Hanna M.D., Mascarenhas M. (2009). Nutrition Management of Pediatric Patients Who Have Cystic Fibrosis. Pediatr. Clin. N. Am..

[106] Ventura A.K., Worobey J. (2013). Early Influences on the Development of Food Preferences. Curr. Biol..

[107] Beauchamp G.K., Mennella J.A. (2009). Early Flavor Learning and Its Impact on Later Feeding Behavior. J. Pediatr. Gastroenterol. Nutr..

[108] Thompson A.L., Bentley M.E. (2013). The Critical Period of Infant Feeding for the Development of Early Disparities in Obesity. Soc. Sci. Med..

[109] Ahern S.M., Caton S.J., Bouhlal S., Hausner H., Olsen A., Nicklaus S., Moller P., Hetherington M.M. (2013). Eating a Rainbow. Introducing Vegetables in the First Years of Life in 3 European Countries. Appetite.

[110] Anzman S.L., Rollins B.Y., Birch L.L. (2010). Parental Influence on Children’s Early Eating Environments and Obesity Risk: Implications for Prevention. Int. J. Obes. (Lond.).

[111] Mennella J.A., Nicklaus S., Jagolino A.L., Yourshaw L.M. (2008). Variety Is the Spice of Life: Strategies for Promoting Fruit and Vegetable Acceptance During Infancy. Physiol. Behav..

[112] Karhunen L.J., Juvonen K.R., Huotari A., Purhonen A.K., Herzig K.H. (2008). Effect of Protein, Fat, Carbohydrate and Fibre on Gastrointestinal Peptide Release in Humans. Regul. Pept..

[113] Paniagua J.A., de la Sacristana A.G., Sanchez E., Romero I., Vidal-Puig A., Berral F.J., Escribano A., Moyano M.J., Perez-Martinez P., Lopez-Miranda J. (2007). A Mufa-Rich Diet Improves Posprandial Glucose, Lipid and Glp-1 Responses in Insulin-Resistant Subjects. J. Am. Coll. Nutr..

[114] Chang K.T., Lampe J.W., Schwarz Y., Breymeyer K.L., Noar K.A., Song X., Neuhouser M.L. (2012). Low Glycemic Load Experimental Diet More Satiating Than High Glycemic Load Diet. Nutr. Cancer.

[115] Zalewski B.M., Patro B., Veldhorst M., Kouwenhoven S., Escobar P.C., Lerma J.C., Koletzko B., Van Goudoever J.B., Szajewska H. (2015). Nutrition of Infants and Young Children (1–3 years) and Its Effect on Later Health: A Systematic Review of Current Recommendations (Earlynutrition Project). Crit. Rev. Food Sci. Nutr..

